# Chitosan Biomaterials for Current and Potential Dental Applications

**DOI:** 10.3390/ma10060602

**Published:** 2017-05-31

**Authors:** Shehriar Husain, Khalid H. Al-Samadani, Shariq Najeeb, Muhammad S. Zafar, Zohaib Khurshid, Sana Zohaib, Saad B. Qasim

**Affiliations:** 1 Department of Dental Materials, College of Dentistry, Jinnah Sindh Medical University, Karachi 75110, Pakistan; shehriarhusain@gmail.com; 2 Department of Restorative Dentistry, College of Dentistry, Taibah University, Al Madinah, Al Munawwarah 41311, Saudi Arabia; MZAFAR@taibahu.edu.sa; 3 Bow River Dental, Calgary, Alberta AB T3B 0M3, Canada; shariqnajeeb@gmail.com; 4 Department of Dental Materials, Islamic International Dental College, Riphah International University, Islamabad 44000, Pakistan; sohail.zafar@riphah.edu.pk; 5 Prosthodontic and Implantology Department, College of Dentistry, King Faisal University, P.O. Box 380, Al-Hofuf, Al-Ahsa 31982, Saudi Arabia; drzohaibkhurshid@gmail.com; 6 Department of Biomedical Engineering, School of Engineering, King Faisal University, P.O. Box 380, Al-Hofuf, Al-Ahsa 31982, Saudi Arabia; szohaib@kfu.edu.sa; 7 Department of Restorative and Prosthetic Dental Sciences, Dar al Uloom University, College of Dentistry, P.O. Box 45142, Riyadh 11512, Saudi Arabia; s.qasim@dau.edu.sa

**Keywords:** natural biomaterials, biopolymers, chitin, dental materials, dental restorations, tissue regeneration

## Abstract

Chitosan (CHS) is a very versatile natural biomaterial that has been explored for a range of bio-dental applications. CHS has numerous favourable properties such as biocompatibility, hydrophilicity, biodegradability, and a broad antibacterial spectrum (covering gram-negative and gram-positive bacteria as well as fungi). In addition, the molecular structure boasts reactive functional groups that provide numerous reaction sites and opportunities for forging electrochemical relationships at the cellular and molecular levels. The unique properties of CHS have attracted materials scientists around the globe to explore it for bio-dental applications. This review aims to highlight and discuss the hype around the development of novel chitosan biomaterials. Utilizing chitosan as a critical additive for the modification and improvement of existing dental materials has also been discussed.

## 1. Introduction

**Figure 1:**
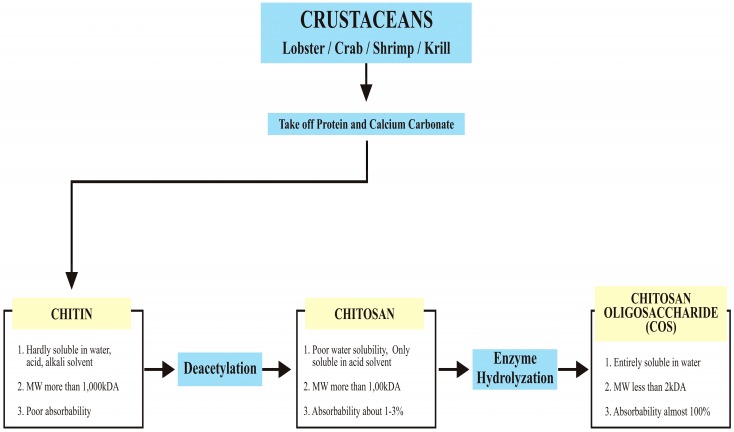
Schematic presentation of deacetylation of chitin derived from crustacean exoskeletons.

Natural biomaterials are known for a range of biological properties such as biocompatibility and biodegradation [1] required for biomedical applications. A few examples of natural biomaterials include collagen [2,3,4] fibrin [5,6,7], natural silk [8,9,10,11], and chitosan [11,12,13,14]. Chitosan is a natural biomaterial that is purified mainly from chitin. The major source of chitin remains the crustacean’s (such as crab and shrimp) exoskeleton [15,16]. Other sources include insects [17,18,19], fungi [18,20] and certain plants such as mushrooms [21,22,23]. During the process of deacetylation (Figure 1), the water-insoluble chitin (Mw > 1000 kDa) changes to chitosan (Mw > 100 kDa) that is poorly soluble in water [24,25]. Further enzymatic hydrolyzation transforms chitosan to chitosan oligosaccharide that has a lower molecular weight (Mw < 2 kDa) and is highly soluble in water [26].

**Figure 2:**
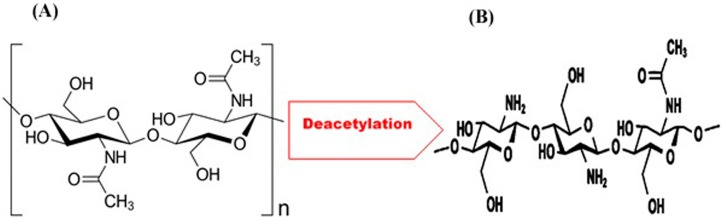
The comparison of chemical structural units: (**A**) chitin; and (**B**) chitosan formed following the process of deacetylation [16].

Chemically, chitosan (CHS) is a polymeric material comprised of *N*-acetylglucosamine and glucosamine copolymer units (Figure 2) [16]. CHS has a range of favourable properties (it is anti-bacterial and biocompatible) and can be combined with various bioactive materials for osteoconductivity [27,28,29]. These unique properties have led to a number of ample opportunities for exploitation in the areas of bioengineering research in general and regenerative medicine in particular [30,31]. For instance, the primary uses of chitosan as a substrate include drug and growth factor delivery [32,33,34], and as a scaffold material for particular types of tissue (bone) engineering [35,36,37]. 

The ability to harness and tailor properties based on particular application gives CHS a significant edge. For example, regarding the material’s properties, the characteristics of chitosan are dependent on structural parameters such as molecular weight and its degree of deacetylation. Moreover, the source of extraction and procedures adapted to conduct deacetylation may affect the final properties. The extent of deacetylation strongly influences physical, chemical and biological properties. It is usually available in low medium and high molecular weights and can be used according to the intended application alone or in composite formulations. Its pH-dependent versatility at a low pH can cause amine groups to be protonated, exhibiting a polycationic nature [38]. At higher pH, chitosan amines are deprotonated and reactive, hence promoting intermolecular interactions advances the formation of fibres, films, porous scaffolds or even gels [12]. Properties such as mechanical strength, biodegradability, and cell affinity can be tailored using various chemical modifications including cross-linking [12,39,40].

**Figure 3:**
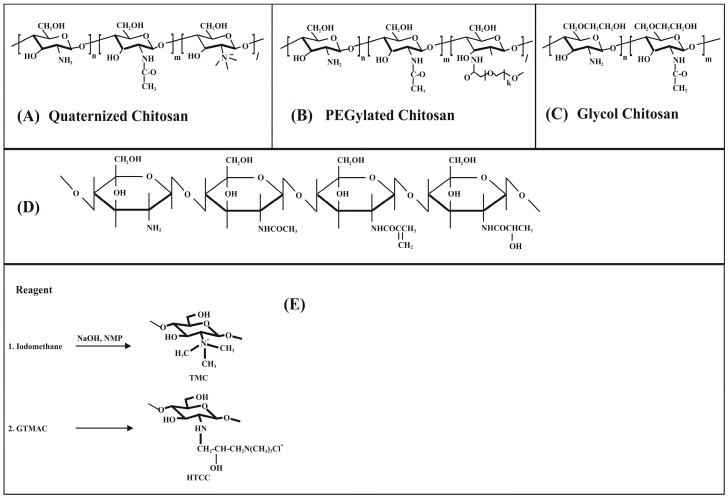
Various structures of modified chitosan in combination with other compounds. (**A**) quaternized chitosan (*N*,*N*,*N* trimethyl chitosan); (**B**) water-soluble polyethylene-glycol conjugated chitosan; (**C**) glycol chitosan containing short ethylene glycol groups [46]; (**D**) water-soluble and cross-linkable chitosan derivative obtained by grafting methacrylic acid and lactic acid onto the pendant amine groups of chitosan [47]; (**E**) quaternized chitosan modified using glycidyl trimethyl ammonium chloride (GTMAC) for protein delivery [48].

Chitosan has been recognised as an antimicrobial agent, however its ability to act in this way is not completely elucidated as several different mechanisms have been attributed to this nature of chitosan [41,42]. One theory suggests that when exposed to bacterial cell wall, chitosan promotes displacement of Ca++ of anionic sites of the membrane, resulting in cellular destruction [43]. It is also known to exhibit a potent antiplaque activity against several oral pathogens such as *Porphyronomas gingivalis*, *Prevotella intermedia* and *Actinobacillus actinomycetemcomitans* [38]. Chitosan has a high degree of biocompatibility in animal models and can be conveniently adapted for the development of implantable biomaterials [44,45]. In addition, chitosan can be chemically functionalized using various compounds (Figure 3).

The design of a suitable dental material is quite a challenging task which hence remains an active area of research as there is still room for improvement in the current commercially available materials [49]. Although there have been some studies documenting the potential or current uses of chitosan in dentistry. To date, no comprehensive reviews have been published reporting applications of chitosan in dentistry. Therefore, the aim of this review is to evaluate the current status of chitosan at the forefront of innovative bio-dental materials development. The potential applications of chitosan are discussed in detail, including the advantages and further prospects.

## 2. Applications of Chitosan Materials in Dentistry

**Figure 4:**
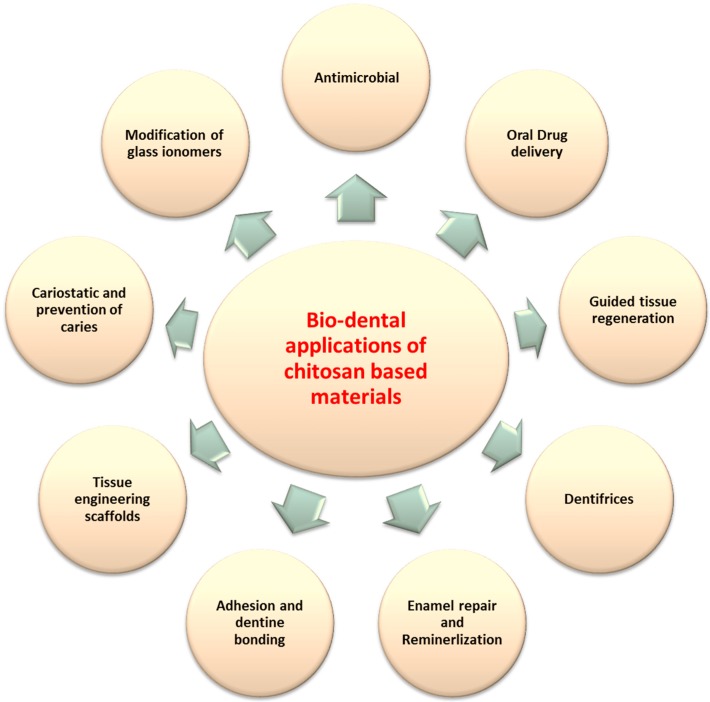
Current and potential applications of chitosan materials in dentistry.

Chitosan has emerged as a potential material for biodental applications corresponding to its unique properties such as bioactivity [50,51], antimicrobial [41,42,52], biocompatibility [14,53,54] and compatibility to blend with other materials [1,55,56]. The chitosan-based materials have been explored extensively for a wide range of dental applications (Figure 4.)

### 2.1. Oral Drug Delivery

Several studies have been conducted to ascertain the potential of chitosan as oral drug carriers [13,57]. Using such drug carriers limits the adverse effects of systemic administration. Chitosan-based composites (CBCs) can be used to design a robust local drug delivery system with the required mechanical properties, contact time, a sustained release profile, while maintaining an intimate contact with the oral mucosa. CBC leads to enhance the bioavailability for treating various oral pathologies. CHS microspheres have been developed for the active release of drugs at sites of pathologies [58,59,60]. Oral administration of CHS is non-toxic. The Food and Drug Administration (FDA) has approved CHS as a food additive. Also, CHS has been explored for drug delivery for a range of biomolecules including DNA, siRNA, growth factors and various drugs [57,61,62,63,64]. The Medical Devices Directive (MDD) has classified all medical devices containing chitin and its derivatives as class III [65].

Chitosan in the form of nano-particles and resorbable films can be used to deliver antibiotics (such as metronidazole, chlorhexidine and nystatin) to periodontal tissues in situ [12,40,66], against fungal infections [33,67] and oral mucositis [33]. The nanoparticles have higher surface area and reactivity to facilitate the drug release [68,69]. Similarly, thiolated chitosan-based formulations have also been used in mucoadhesive patches to prevent dental caries. The sustained release of antibacterial medicament inhibits the growth of cariogenic *Streptococcus mutans* [28,70]. Although electrospun mats display useful properties such as surface smoothness and non-toxicity coupled with a rapid release of the incorporated substances [14,71,72], further studies are required to validate the exact nature of the release profile and whether the sustained release of drugs can be maintained [12,42,70,73]. 

The adhesion of oral bacteria to the tooth surfaces (e.g., hydroxyapatite (HA)) can be considered for the synthesis of antibacterial medicaments, and dentifrices for the oral environment. Chitin and chitosan have long been implicated with respect to their bacteriostatic and bactericidal actions against a variety of oral microorganisms. The roles of *S. mutans* [74,75,76] and *Porphyromonas gingivalis* [77,78] have been recognised in dental caries and periodontal disease, respectively. On the whole, chitosan materials have low toxicity and antimicrobial activity levels ranging from 100 to 100,000 mg L^−1^ and 100 to 1250 mg L^−1^ against gram-negative and gram-positive bacteria, respectively [41]. It has been established that low molecular weight chitosan varieties have a profound role towards impeding colonization of pathogenic strains (such as *S. mutans*) on tooth surfaces without disrupting normal oral flora [79]. Chitosan ranging from low molecular weight (50–190 kDa), medium molecular weight (190–300 kDa) and high molecular weight (310–375 kDa) may be implicated at some level in terms of imparting antimicrobial activity [80]. For instance, low molecular weight chitosan has superior penetrating capabilities, hence impairing the bacterial physiological activities at the cellular level. In contrast, high weight molecular chitosan is credited for an indirect approach involving the formation of a film around the bacterial cells and choking the entry of nutrients to the central metabolic machinery [81,82]. 

A comprehensive understanding of the antibacterial activity of CHS-based materials remains elusive to date. Plausible theories have been represented with respect to the protonation of the amino groups of CHS upon coming in contact with physiological fluids such as saliva. The cationic species thus generated interact with the anionic groups on the bacterial cell wall, imparting a makeshift bacteriostatic effect by bacterial agglutination and/or alterations in permeability—an impediment to uncontrolled growth [83]. Other investigators have implicated the Ca++ displacement from the membranes upon interaction with chitosan as a plausible alternative [43]. Camacho et al. 2010 [84] described antimicrobial properties stemming from the positive charge possessed by the CHS polymeric chain amino groups counteracting with negatively charged macromolecular remnants e.g., proteins and lipopolysaccharides in the cell membrane. This eventually leads to obstruction of nutrient exchange between the cell interior and the extracellular matrix. The electrostatic charges are responsible for competing for calcium for electronegative sites in the membrane, hence compromising the integrity. This process leads to the subsequent release of intracellular material and cellular death. In dentistry, CHS has displayed effective plaque control in vitro by inhibiting specific dental plaque pathogens (*Actinobacillus actinomycetemcomitans, P. gingivalis* and *S. mutans*) [27,28].

### 2.2. Guided Tissue Regeneration (GTR)

**Figure 5:**
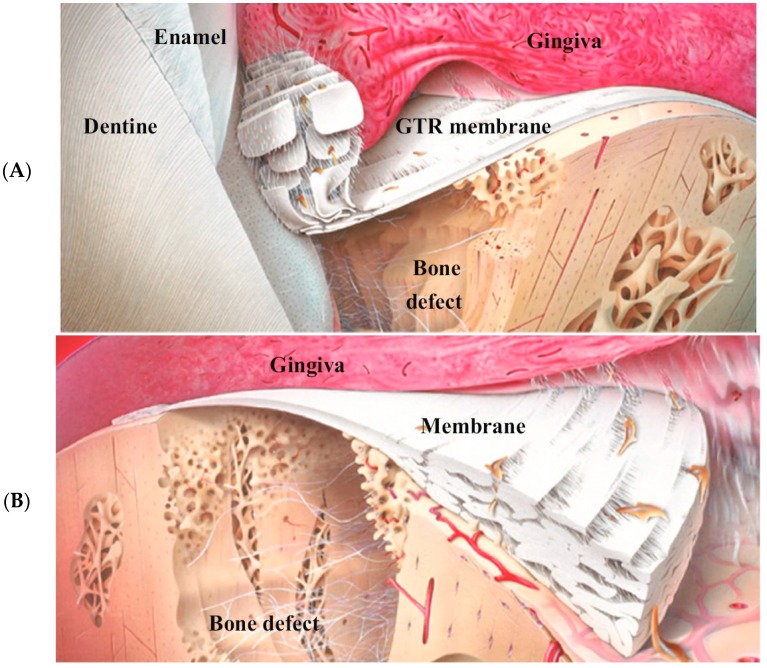
The schematic presentation of bone regeneration using the guided bone regeneration (GBR) approach. (**A**) shows the barrier preventing the contact of “the dentogingival epithelium and gingival connective tissues” with the curetted root surface; (**B**) shows the Gore-Tex augmentation membrane in a closed (primary soft tissue coverage) supporting new connective tissue regeneration and attachment on a previously periodontaly involved root surface (adapted from Scantlebury and Abmbruster [98] with the permission from publisher). GTR: guided tissue regeneration

Periodontal disease is considered a major affliction worldwide [85]. The growing world population indicates a predictable increment in periodontal diseases. This can be correlated with an increase in the average age and centurions globally [86,87]. Periodontitis is a chronic inflammatory process that culminates irreversible loss of periodontal tissues and ultimately tooth loss [85]. The inhospitable mouth environment becomes worse in the presence of periodontitis [49]. A variety of periodontal treatment approaches rely mostly on oral hygiene maintenance, plaque control [88,89] and direct localised clinical/surgical intervention for promoting healing of the periodontal tissues [90,91]. There is an increasing level of interest in developing regenerative periodontal therapeutic strategies vis-à-vis the concept of guided tissue regeneration (GTR) or guided bone regeneration (GBR) [92,93,94]. The technique of GBR has been shown schematically in Figure 5. This relatively novel therapeutic modality has been at the centre of numerous successful clinical trials [95,96]. The underlying strategy in GTR involves isolating the periodontal defect with a suitable membrane (resorbable or non-resorbable) that acts as a physical impediment to gingival tissue infiltration into the osseous defects, thereby encouraging bone regeneration and preventing spaces for fibrous tissue proliferation simultaneously [97]. 

In order to achieve this objectively in an efficient and clinically viable manner, it is imperative for the template to possess certain biological, physical, chemical and bioactive characteristics that encourage favourable host tissue response in a self-contained temporal system amenable to tissue regeneration [99,100]. The spectrum of properties desirable in a comprehensive GTR membrane therapy system ranges from robust constructs (smart, bio-integrative and conducive) to drug delivery applications. An optimal particle size and biological behaviour of the inclusive elements improves the receptiveness to cellular and extracellular matrix (ECM) cues. Due to the compliance with the aforementioned properties, chitosan has been pinned as a favourable substrate material for periodontal tissue regeneration. Some investigators have worked on producing and subsequently analysing chitosan membranes coated with a bioactive material such as a bioceramic-based agent like HA and calcium phosphate variants which include tricalcium phosphate (TCP) α and β [101]. Other researchers also looked into building on the promising results of the former by the addition of a cross-linking agent such as sodium tripolyphosphate [102], genipin [103,104] and glutaraldehyde. This was done as a pretext to enhancing the mechanical properties (such as modulus of elasticity, hardness and toughness) of the chitosan membrane substructures. Composite formulations of chitosan and hydroxyapatite have been heavily investigated to fabricate chitosan and HA templates using novel methodologies [105,106,107]. Ang and co-workers [107] deposited layer by layer chitosan–HA composite materials using a preprogrammed lay-down pattern and a desktop rapid prototyping system. Chavanne and colleagues also worked on similar lines and developed a porous cylindrical template [108].

A number of studies have proposed the concept of functionally graded membranes [12,61,109]. The concept revolves around employing the use of a graded structure at the defect site around the tooth and/or implant interface. This approach fully addresses the local biological, physicochemical and functional requirements for the functioning of functionally graded membranes (FGMs) in situ [97,110,111]. The syntheses of functionally graded membranes using different material fabrication protocols have been reported. These include the use of layered casting protocols comprising PLGA, nanohydroxyapatite and collagen [112] with an HA/collagen/PLGA porous side for promoting adequate levels of resident cellular recruitment and adhesion. Other uses involve electrospinning techniques for fabricating graded nanofiber scaffolds and multilayer electrospinning [113] for designing FGMs with a stabilising core constituting poly L lactide co-caprolactone and two functionalized surface layers of HA and a polymer–gelatine composite [97]. The grading of structures by such a method provides the means to tailor the time stability and further improve the periodontal outcome [49]. Recently, Qasim et al. [113] have reported the development and subsequent characterisation of porous CHS–HA membranes using ascorbic acid and acetic acid as solvents. The freeze gelation technique was used with the aim of developing a suitable core layer boasting desirable mechanical and biological properties in an FGM construct for periodontal tissue regeneration.

Regardless of other components (cross-linking agents and bioactive calcium phosphates), the formation of a bio-ceramic layer of variable crystallinity was detected on templates with a chitosan backbone. The functionally graded conditions are essential for tissue implant interface. There are three possible perspectives; biological, mechanical and anatomical. In terms of biological perspective, one layer may comprise cell bearing phenotypes that may differ to other layers within the construct hence influencing the quality and distribution of ECM production. Considering mechanical properties, the scaffolds should closely match those of the target tissues. This would be synchronous with the intention of producing a template that would be devoid of localised stress concentration regions along its entire covered area. Moreover, the resident cells would receive similar mechanical cues as in a physiological environment [114].

### 2.3. Modifications of Dentifrices

Toothpastes are known to be an integral component of the daily oral hygiene maintenance regimen. Their role is evident in warding off dental erosive demineralisation of the tooth structure due to intermittent exposure to acidic drinks. Many toothpaste formulations and their modifications have been investigated over the years with different active ingredients (Table 1). These include preparations containing nanohydroxyapatite, 5% KNO_3_ [115,116,117] and SnF_2_ [118,119] with the intent of complementing the action of NaF towards remineralisation and re-hardening of enamel surfaces. Although the literature gives mixed results with respect to the anti-erosive effect of the aforementioned additives, Sn-containing dentifrices were deemed the most efficient in terms of imparting excellent anti-erosive potential compared to standard NaF-based formulations.

Ganss et al. [120] reported on the commercially available chitosan-based dentifrice (Chitodent^®^ (B&F)), that is a non-fluoride formulation, and highlighted a significant reduction of tissue loss. Similar results have been reported while using NaF- and Sn-based dentifrices, i.e. with respect to hindering erosion of the dentin organic matrix [124] and enamel. These findings can be attributed to the cationic nature of chitosan coupled with a low pH, and high affinity for binding to structures with negative zeta potentials such as enamel and salivary pellicles. This would result in the subsequent formation of a protective multilayer organic matrix over mineralized surfaces [127,128]. In a similar setting, a chitosan additive enhanced the efficacy of Sn^2+^-based dentifrices towards tackling tissue loss in acidic oral environments by imparting dual-pronged anti-erosive and anti-abrasive effects [122,129].

### 2.4. Enamel Repair

Tooth enamel is a non-vascular and the hardest tissue of human body [130,131] hence the repair or regeneration of enamel is challenging. A number of chitosan-based restorative formulations have been explored and are under consideration for achieving human enamel regeneration through successful delivery of organic amelogenin at the site of enamel defects. Recently, Ruan et al. [132] employed a chitosan-based hydrogel as a delivery medium for amelogenin with the aim of rejuvenating the aligned crystal structure. The use of chitosan imparts a dual effect of offering a protective effect against secondary caries corresponding to its antibacterial properties along with not influencing enamel crystal orientation [28,70,133]. More research involving disciplines such as tissue engineering, biomolecules and materials science is required to explore the further potential of chitosan for enamel regeneration applications.

### 2.5. Adhesion and Dentine Bonding

The dentine-restoration interface and durability of bond strength have captured the interest of researchers. Currently, the dentine replacement materials have issues such as technique sensitivity of acid etching and removal of the smear layer [134]. The incomplete removal of the smear layer often gives rise to poor penetration of the resin monomer resulting in an unstable hybrid layer that is prone to nano leakage [135]. Hence, the area of bioadhesive polymers in general and chitosan-based dentine replacement materials in particular merits special attention. Antioxidant chitosan hydrogels with propolis, β carotene and nystatin were investigated and translated significant grounds towards delivering robust dentine bonding systems with a concomitant increase in shear bond strength. Some formulations tested in the studies reported shear bond strength values of up to 38 MPa after 24 h [136] and in excess of 20 MPa after 6 months. These values were deemed to be significantly higher than conventional dentine bonding systems with or without phosphoric acid treatment [137].

### 2.6. Modification of Dental Restorative Materials

There has been a significant level of effort towards paving the way for the entry of novel biomimetic dental restorative materials for clinical applications. The extent of damage to the enamel and/or components of the pulp/dentin complex are very significant in terms of promoting and treatment prognosis [138,139,140]. However, some of the drawbacks associated with bioactive restorative materials currently in development include poor adhesion coupled with less than desirable mechanical properties compared to resin and ceramic-based restorative materials. These discrepancies result in the interfacial failure owing to a mismatch of physical and chemical properties [141,142,143,144]. Among restorative materials, glass ionomers (fluoroaluminosilicate glass powder with poly(acrylic) acid liquid) form a chemical adhesion with the calcified tooth tissues [145]. Glass ionomer cements (GICs) have been presented with various modifications such as with resin or nano-additives [146,147,148] and are commonly used for applications such as cementation of prosthesis and restorations. 

The favourable physicochemical properties of GICs, such as antibacterial effects and sustained fluoride release [149,150], biocompatibility, and superior natural affinity for tooth structure (enamel, dentine) reduce instances of microleakage or interfacial failure [146,151,152,153]. On the other hand, GICs are associated with insufficient fracture toughness, and flexural strength, particularly in the case of bulk-filled restorations. Hence conventional GICs are mostly associated with inferior mechanical properties, especially when used to replace lost tooth material in high load bearing areas [151,154,155]. The role of chitosan as a biocompatible polysaccharide oriented towards enhancing mechanical properties of GICs has been the subject of some featured investigations. These include the works of Petri et al. [156] who came up with much-improved values of the flexural strength of GICs post addition of chitosan with the added benefit of increasing the rate at which fluoride ions leached from the set material (Table 2). In addition, small volumes of CHS added to GICs (10% w/v) impart no adverse effects towards their performance in terms of microleakage, which paves the way for it to be a viable and promising candidate as an additive to GICs [157].

The facilitating role of CHS in the way of enhancing protein release profile when added to GICs is attributed to the formation of polymer complex phases as a result of the interaction of the CH with poly(acrylic) acid [158,159]. The concept has been taken a step further with respect to dental pulp regeneration. CHS has been added to conventional GICs with the aim of evaluating its effect on protein and/or growth factor release in the build-up to achieving reliable methods of vital pulp therapy [140,160]. Limapornvanich et al. [161] studied the release profile of bovine serum albumin (BSA) from CHS-modified GICs. The results indicated no cytotoxic effect on pulp cells coupled with a prolonged release effect of BSA, relative to conventional glass ionomer cement, when in contact with this formulation. These findings are suggestive of the biocompatible nature of CHS, and the interaction of its -NH_2_^+^ cationic and the anionic groups of poly(acrylic acid) towards the formation of complexes, respectively [158,162]. There have been attempts to catalogue the synergistic effect of CH and albumin in resin-modified GICs supplemented with translationally controlled tumour protein (TCTP) [163] and transforming growth factor beta-1 (TGF β1) [163,164] with respect to attaining a lower cell cytotoxicity value and the simultaneous promotion of anti-apoptotic activity in pulp cells as a pretext to promoting remineralization. This could be a significant development in the way of developing dental pulp-friendly restorative materials that offer a shielding effect from the toxicity stemming from the residual acid and monomer 2-hydroxyethyl methacrylate (HEMA) components of GICs and the resin element, respectively. The findings were in line with earlier reports regarding the leached HEMA monomer—a necessary component required to improve mechanical properties such as elastic modulus, flexural strength and wear resistance to name a few [165], as the primary culprit responsible for inducing pulp cell apoptosis [166,167,168]. Therefore, CHS-modified GICs could have applications in the area of bioactive dental restorations and regenerative endodontics in the guise of a vital pulp therapy material. Some restrictions to be taken into account with respect to in vitro studies assessing protein release from CH modified GICs implicate loss of some chemical extracts when filtering the residue prior to an MTT assay evaluation for cell cytotoxicity. 

### 2.7. Chitosan for Coating Dental Implants

The clinical success of dental implants is based on degree of osseointegration of implant materials and alveolar bone [169,170,171]. In order to improve the osseointegration, a number of surface treatment and implant coatings have been tried, with promising results [172,173,174,175]. In addition, bioactive coatings of dental implants showed improved clinical longevity in medically compromised patients, affecting their bone health [169,173,176,177]. A number of studies have reported promising results for chitosan coating of dental implants [31,178,179]. The chitosan coating may affect the surface and bone interface by altering biological, mechanical and morphological surface properties. For example, considering mechanical properties, chitosan coating changes the elastic modulus hence reducing the mismatch between the implant surface and alveolar bone and reducing the areas of stress concentration [114]. Moreover, the chitosan coatings can potentially be used to carry various medicaments such as antibiotics for localised delivery around the implant area. However, further research is required to validate either such coatings are beneficial to inhibit infection and promote the osseointegration [180].

### 2.8. Stem-Based Regenerative Therapeutics

Stem cell-based transplantation strategies hold a great potential in the field of dentistry and can revolutionise the approach to treat diseases and alleviate oral conditions using embryonic stem cell (ESCs), and more recently adult dental stem cells, to induce pluripotent stem cells (iPSC) in tooth regeneration [93,181]. Moreover, rapid advancements in this field have led to the use of chitosan as a carrier for chitosan-mediated stem cell repair. The regeneration of dentine-pulp complex has been investigated by exploiting the regenerative potential of mobilised dental pulp stem cells (MDPSc) by Nakashima and co-workers in a clinical trial. The demonstrated that human MDPSc is safe and efficacious for complete pulp regeneration in the pilot study [182,183]. Yang et al. reported the use of dental pulp stem cells cultured on a collagen–chitosan complex and were also able to form a dentine–pulp complex [184]. The regeneration of entire tooth is also expected to be a goal of current research clusters. Tooth engineering to form dental structures in vivo has been established using different stem cells. Moreover, stem cell technology for regenerative therapies is already available as mesenchymal stem/ stromal cells (MSCs) already have been introduced in the clinic for alveolar bone augmentation [185,186].

## 3. Conclusions

Chitosan is a new biomaterial for dental applications ranging from restorative dentistry to tissue engineered scaffolds for the alveolar bone to periodontal complex healing. Although it has gone through rigorous investigations for its biocompatible nature, antimicrobial properties, and adjustable degradation characteristics according to the application, there still remain certain issues that need addressing, such as the fact that extracted chitosan may vary in terms of structure and molecular weights from low, medium to high, resulting in inconsistent physiochemical characteristics and variability. These variations, especially when looking at dental applications, are an issue as the molecular weight range varies, and reproducibility of the correct molecular weight is still a challenging task. Nevertheless, this ever-evolving field of dentistry can use this naturally derived polymer to its advantage in numerous other prosthetic, orthodontic and implant-related fields as well. Therefore, there is excellent potential for expanding its biological applications in future. However, very little clinical data is available regarding the clinical dental applications of chitosan-based materials. In order to translate chitosan-based materials from research to clinical applications, there is need for further research, particularly in vivo studies and clinical trials.

## Figures and Tables

**Table 1:** Studies reporting effects of chitosan-modified dentifrices.

Study	Type of Study	Active Ingredients of Tested Dentifrice	Controls	Erosive Solution (s)	Methodology	Results
Ganss et al. [120]	In vitro	Chitosan, NaF, KNO_3_/NaF, HA/NaF, ZnCO_3_-HA, SnF_2_	F-free mouthwash, F-containing mouthwash	0.05 M citric acid	Profilometric analysis of extracted teeth; immersion only and brushing	Slurry only: SnF_2_ most effective (*p* ≤ 0.005); Toothbrush simulation: KNO_3_ most effective (*p* ≤ 0.005).
Ganss et al. [121]	In vitro	NaF, NaF/SnCl_2_, AmF/NaF/SnCl_2_, AmF/NaF/SnCl_2_/chitosan, AmF/SnF_2_,	SnF_2_, placebo toothpaste	0.05 wt. % citric acid	Profilometric analysis of extracted teeth; brushing	AmF/NaF/SnCl_2_/chitosan was most effective in preventing tissue loss (*p* ≤ 0.01).
Schlueter et al. [122]	Random-ised in situ trial (double blinded)	F/Sn, F/Sn/chitosan	Placebo toothpaste, SnF_2_ gel	0.5% citric acid	Profilometric analysis of enamel specimens in situ; slurry (3 weeks) without/with brushing	No significant difference among Sn-containing pastes after only immersion and immersion and brushing.
Ozalp et al. [123]	In vitro	Chitosan, propolis, AmF	No treatment	Demineralization solution	SEM-EDX analysis of sound and demineralized brushed enamel	No significant differences between the tested pastes on sound lesions.
Ganss et al. [124]	In vitro	NaF, AmF/NaF/SnCl_2_/chitosan	Placebo, SnF_2_ gel	Citric acid (1%), citric acid (1%) + collagenase	Profilometric analysis of dentine sections; slurry only, slurry + brushing	AmF/NaF/SnCl_2_/chitosan significantly reduced erosion with organic tissue loss when brushed (*p* ≤ 0.05). No differences with slurries only.
Carvalho and Lussi [125]	In vitro	NaF (with and without NaF rinse), F/Sn/chitosan (with and without Sn rinse)	Placebo toothpaste	Artificial saliva, 1% citric acid	SEM/EDX of enamel specimens brushed with tested toothpastes Surface micro-hardness, tooth structure loss	F/Sn/chitosan followed by Sn rinse showed the least reduction in surface hardness (*p* < 0.001) and the least substance loss (*p* < 0.05).
Aykut-Yetkiner et al. [126]	In vitro	AmF, NaF/Nano-HA, ZnCO_3_-HA, NaF/AmF/SnCl_2_/Chitosan, NaF/HA, NaF/KNO_3_	No treatment	Citric acid, HCl/pepsin	Profilometry of bovine dentine specimens brushed with tested toothpastes	All toothpastes reduced significantly but AmF toothpaste had the most significant effect.

Amine fluoride (AmF); hydroxyapatite (HA); potassium nitrate (KNO_3_); sodium fluoride (NaF); scanning electron microscope elemental analyses (SED-EDX); zinc carbonate (ZnCO_3_), stannous fluoride (SnF_2_).

**Table 2:** Summary of the flexural strengths of different formulations of chitosan-modified glass ionomer restorations along with estimated fluoride release [156]. GICs: glass ionomer cements.

Chitosan in GICs (wt. %)	Flexural Strength (MPa)	Fluoride Release (µg/cm^2^)	
		After 21 h	After 1 Month
0	14.27 ± 2.60	~100	~500
0.004	18.41 ± 3.26	~1500	~3700
0.012	17.00 ± 3.98	~400	~1000
0.025	15.07 ± 4.34	NR	NR
0.045	6.88 ± 1.63	NR	NR
